# Effects of serum amyloid A on the structure and antioxidant ability of high-density lipoprotein

**DOI:** 10.1042/BSR20160075

**Published:** 2016-08-16

**Authors:** Megumi Sato, Ryunosuke Ohkawa, Akira Yoshimoto, Kouji Yano, Naoya Ichimura, Madoka Nishimori, Shigeo Okubo, Yutaka Yatomi, Minoru Tozuka

**Affiliations:** * Analytical Laboratory Chemistry, Field of Applied Laboratory Science, Graduate School of Health Care Sciences, Tokyo Medical and Dental University, 1-5-45 Yushima, Bunkyo-ku, Tokyo 113-8519, Japan; † Department of Clinical Laboratory, The University of Tokyo Hospital, 7-3-1 Hongo, Bunkyo-ku, Tokyo 113-8655, Japan

**Keywords:** antioxidant ability, high-density lipoprotein, inflammation, serum amyloid A

## Abstract

Serum amyloid A (SAA) levels increase during acute and chronic inflammation and are mainly associated with high-density lipoprotein (HDL). In the present study, we investigated the effect of SAA on the composition, surface charge, particle size and antioxidant ability of HDL using recombinant human SAA (rhSAA) and HDL samples from patients with inflammation. We confirmed that rhSAA bound to HDL_3_ and released apolipoprotein A-I (apoA-I) from HDL without an apparent change in particle size. Forty-one patients were stratified into three groups based on serum SAA concentrations: Low (SAA ≤ 8 μg/ml), Middle (8 < SAA ≤ 100 μg/ml) and High (SAA > 100 μg/ml). The ratios of apoA-I to total protein mass, relative cholesterol content and negative charge of HDL samples obtained from patients with high SAA levels were lower than that for samples from patients with low SAA levels. Various particle sizes of HDL were observed in three groups regardless of serum SAA levels. Antioxidant ability of rhSAA, evaluated as the effect on the formation of conjugated diene in low-density lipoprotein (LDL) induced by oxidation using copper sulfate, was higher than that of apoA-I. Consistent with this result, reconstituted SAA-containing HDL (SAA-HDL) indicated higher antioxidant ability compared with normal HDL. Furthermore, HDL samples obtained from High SAA group patients also showed the highest antioxidant ability among the three groups. Consequently, SAA affects the composition and surface charge of HDL by displacement of apoA-I and enhances its antioxidant ability.

## INTRODUCTION

Serum amyloid A (SAA) is one of the acute phase proteins predominantly produced in the liver during acute phase response (APR) to inflammatory stimulus such as infection, trauma, surgery and malignant tumour [1]. SAA is also modestly induced by chronic inflammatory disorders including obesity, metabolic syndrome, type 2 diabetes and rheumatoid arthritis [2]. SAA is well known as a precursor of AA amyloidosis, and many studies have been focused on the pathological mechanisms involved in this disease [3]. However, the physiological function of SAA has not been clearly determined yet. SAA, a polypeptide composed by 104 amino acids, consists of four isoforms, SAA1–SAA4 [4]. SAA1 and SAA2 are major acute phase isoforms synthesized in hepatic cells [5]. SAA3, a minor isoform considered a pseudogene, was recently found to be expressed as a fusion protein with SAA2 in humans [6]. SAA4 is a constitutive isoform that represents more than 90% of total SAA under normal physiological conditions [7]; however, the level does not change dramatically in response to inflammation [8]. A recent structural investigation indicates that an amphipathic α-helical structure in the N-terminal region of SAA is important for lipid interaction [9]. SAA circulates in plasma as a component of one of the lipoproteins, high-density lipoprotein (HDL) [10].

HDL-cholesterol (HDL-Cho) levels have shown inverse correlation with the risk of coronary heart disease (CHD) in clinical and epidemiological studies [11]. HDL performs reverse cholesterol transport (RCT), transferring excess cholesterol from peripheral tissues to the liver for subsequent excretion into the bile and faeces. This central role of HDL is regarded as the most important function for HDL-mediated atheroprotection [12]. Moreover, HDL has several anti-atherogenic properties such as inhibition of low-density lipoprotein (LDL) oxidation, promotion of endothelial repair, improvement of endothelial function, anti-thrombotic function and anti-inflammatory ability [13]. Apolipoprotein A-I (apoA-I), the primary protein component of HDL, is considered to play an essential role in reducing the progression of atherosclerosis [14].

During APR, SAA is induced by proinflammatory mediators such as interleukin 1 (IL-1), interleukin 6 (IL-6) and tumour necrosis factor α (TNF-α) [4]. Therefore, the concentration of circulating SAA increases up to 1000-fold over the basal level (<8 μg/ml) [1]. Reportedly, when SAA was released from the liver, it bound to HDL followed by a displacement of apoA-I [1,15,16]. In contrast, another report indicated that SAA generated SAA-containing HDL (SAA-HDL) through ATP-binding cassette transporter A1 (ABCA1) in the liver, in a similar way to the biogenesis of normal HDL by apoA-I [17]. As stated above, there is a few reports that have shown the displacement of apoA-I by SAA, however, the most of these reports have demonstrated the displacement using mouse materials (HDL and recombinant SAA), not human materials [15,16]. In another study, although human HDL and recombinant human SAA (rhSAA) were used, free SAA was not removed from the mixture of HDL and rhSAA after the incubation [1]. Therefore, it was unclear whether the SAA actually combined with HDL or aggregated and formed particles sized similar to HDL. It is necessary to determine the direct impact of SAA on HDL features.

In the present study, we evaluated the structural change of HDL induced by SAA during inflammation. We also examined the functional change of HDL, especially relative to its antioxidant ability, associated with the structural change, using reconstituted SAA-HDL and HDL obtained from the patients with high SAA levels in serum.

## MATERIALS AND METHODS

### Chemicals

All chemicals were from Wako Pure Chemical Industries if not stated otherwise.

### Serum samples

Serum samples of the patients were obtained from the clinical laboratory of The University of Tokyo Hospital. The patients were stratified into three groups based on the concentration of serum SAA: Low (SAA ≤ 8 μg/ml, *n*=11), Middle (8 < SAA ≤ 100 μg/ml, *n*=10) and High (SAA > 100 μg/ml, *n*=20). Normal serum samples were obtained from apparently healthy volunteers from the Graduate School of Health Care Sciences, Tokyo Medical and Dental University. The study was approved by both, the Institutional Research Ethics Committee of the Faculty of Medicine, The University of Tokyo, and the ethics committee of the Faculty of Medicine, Tokyo Medical and Dental University.

### Isolation of lipoproteins

LDL (*d*=1.006–1.063 g/ml) and HDL (*d*=1.063–1.210 g/ml) were isolated by ultracentrifugation as described previously [18]. Isolated LDL and HDL from healthy volunteers were dialysed against PBS. HDL from patients was applied to PD-MiniTrap G-25 (GE Healthcare) chromatography columns to eliminate potassium bromide (KBr). Lipoproteins were stored at −80 or 4°C until used for the antioxidant ability assay or other experiments, respectively.

### Preparation of reconstituted SAA-HDL

HDL isolated from normal serum was mixed with rhSAA (Eiken Chemical) at the final concentration of 25 mg/dl HDL-Cho and 1 mg/ml SAA, mimicking the ratio present in the APR state [19]. The mixture was then incubated at room temperature for 60 min. To remove free rhSAA, the density of the mixture was adjusted to 1.210 g/ml and ultracentrifuged again. The top layer including reconstituted SAA-HDL was applied to a PD-MiniTrap G-25 chromatography column to eliminate KBr.

### Electrophoresis and western blotting

Agarose gel electrophoresis for lipoprotein fractionation was performed using a commercial kit (Helena) following the manufacturer's protocol, and the separated lipoproteins including HDL were stained with 0.1% Fat Red 7B. Western blot analysis was performed as described previously [10]. Briefly, the samples were subjected to SDS/PAGE using 14% acrylamide gel under non-reducing conditions, and to non-denaturing gel electrophoresis using 8% acrylamide gel or 4–20% gradient acrylamide gel (TEFCO). Proteins separated by electrophoresis were stained with CBB or transferred to PVDF membranes (Millipore). PVDF membranes were incubated with goat anti-apoA-I polyclonal antibody (Academy Bio-Medical Company) and peroxidase (POD)-conjugated rabbit anti-goat IgG (Medical & Biological Laboratories) for apoA-I detection, and with rabbit anti-SAA polyclonal antibody (ASSAYPRO) and POD-conjugated goat anti-rabbit IgG (Beckman Coulter) for SAA detection. Finally, the bands containing apoA-I and SAA were visualized with 3,3′-diaminobenzidine-4HCl and H_2_O_2_ or with ECL Prime Western Blotting Detection Reagent (GE Healthcare).

### Antioxidant ability

Antioxidant ability of rhSAA and apoA-I was evaluated as described previously [20]. Fifty μg protein/ml LDL obtained from pooled serum was incubated with 1 μM copper sulfate in the presence or absence of 10 μg/ml rhSAA, 10 μg/ml apoA-I. LDL oxidation was monitored as the formation of conjugated dienes at 234 nm in 10 min intervals. Antioxidant ability was defined as relative prolongation of the lag time and as relative decrease in the maximum velocity (*V*_max_) compared with LDL alone (Supplementary Figure S1). In the same manner, we compared antioxidant ability of 50 μg protein/ml reconstituted SAA-HDL with normal HDL. The antioxidant ability of HDL was determined for samples obtained from the patients in each group (Low, Middle and High).

### Measurements of SAA and apoA-I

SAA levels were measured by latex agglutination-turbidimetric immunoassay with commercially available kit; LZ test ‘Eiken’ SAA (Eiken Chemical). ApoA-I concentration was determined by turbidimetric immunoassay using commercial kit; ‘apoA-I auto N ‘Daiichi’ (Sekisui Medical). Both SAA and apoA-I assay were performed using an automatic analyser, JCA-BM8000 series (JEOL), as following the manufacturer's protocol.

### Other measurements

The concentration of total protein (TP) was measured by Lowry method [21]. Total cholesterol (TC), triacylglycerol (TG) and phospholipid (PL) levels were measured using enzymatic methods with the corresponding commercial kits (Kyowa Medex).

### Statistical analysis

Data are expressed as the mean ± S.D. The independent or paired *t* test was used to evaluate differences between rhSAA and apoA-I. Comparison among the three groups was performed using Kruskal–Wallis test. Spearman rank correlation test was employed to determine the significance of the correlation between serum SAA levels and lag times obtained from the antioxidant ability assay. Three stages of *P* values (less than 0.05, 0.01 and 0.001) were considered statistically significant.

## RESULTS

### HDL remodelling induced by rhSAA

**Figure 1:**
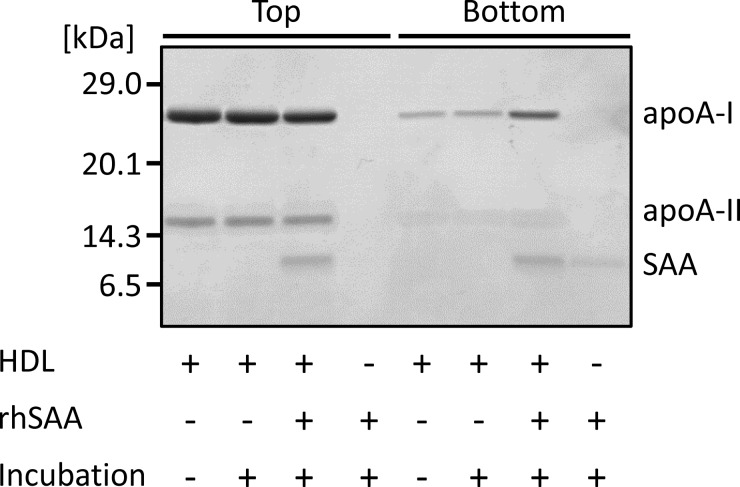
Displacement of apoA-I by SAA rhSAA (final concentration of 1 mg/ml) was incubated with HDL obtained from healthy subjects (final HDL-Cho of 25 mg/dl) for 60 min at room temperature. The mixture was ultracentrifuged at the density of 1.210 g/ml. Top and bottom fractions were analysed by SDS/PAGE and visualized by CBB staining. The figure is representative of four independent experiments.

**Figure 2:**
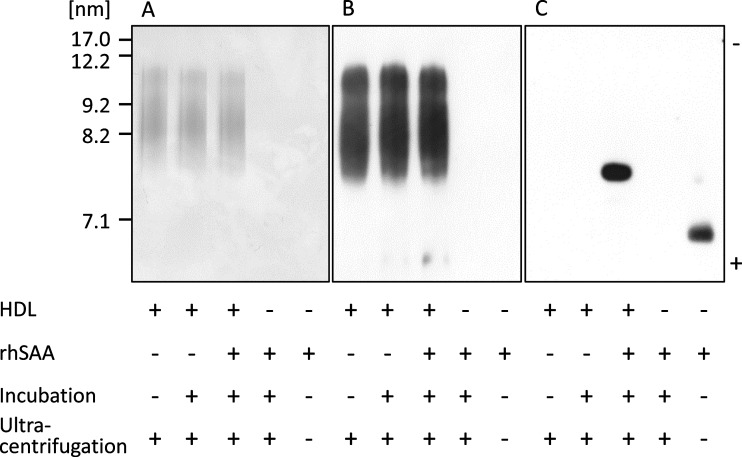
Features of HDL remodelled by SAA rhSAA (final concentration of 1 mg/ml) was incubated with HDL obtained from healthy subjects (final HDL-Cho of 25 mg/dl) for 60 min at room temperature. The mixture was ultracentrifuged at the density of 1.210 g/ml. Top fraction was analysed by non-denaturing gel electrophoresis and visualized by CBB staining (**A**) and western blotting using anti-apoA-I antibody (**B**) and anti-SAA antibody (**C**). The figure is representative of four independent experiments.

To test the effect of SAA on HDL structure, rhSAA was incubated with normal HDL at a ratio similar to that in the APR state, and after ultracentrifugation, HDL fractions (Top) and protein fractions (Bottom) were analysed by SDS/PAGE and non-denaturing gel electrophoresis. For the top layer of normal HDL with or without incubation with rhSAA, two bands at apparent molecular masses of 28 and 16 kDa corresponding to apoA-I monomer and apoA-II dimer, respectively, were detected (Figure 1). Faint bands of apoA-I were detected for the bottom layer due to its release by physical force during ultracentrifugation. In contrast, the band at 11.7 kDa for rhSAA was detected for the top layer, after incubation with rhSAA. Uncombined rhSAA and apoA-I were also observed for the bottom layer; however, the intensity of apoA-I band was obviously higher than that of HDL untreated with rhSAA (Figure 1), indicating the displacement of apoA-I by rhSAA. By non-denaturing gel electrophoresis, rhSAA did not markedly affect HDL particle size and apoA-I distribution (Figures 2A and 2B). In contrast, a sharp band of rhSAA was observed for the HDL fraction at the position of the smallest HDL_3_, previously described as HDL3c (7.2–7.8 nm) [22] (Figure 2C).

### Features of HDL from the patients with various SAA levels

**Figure 3:**
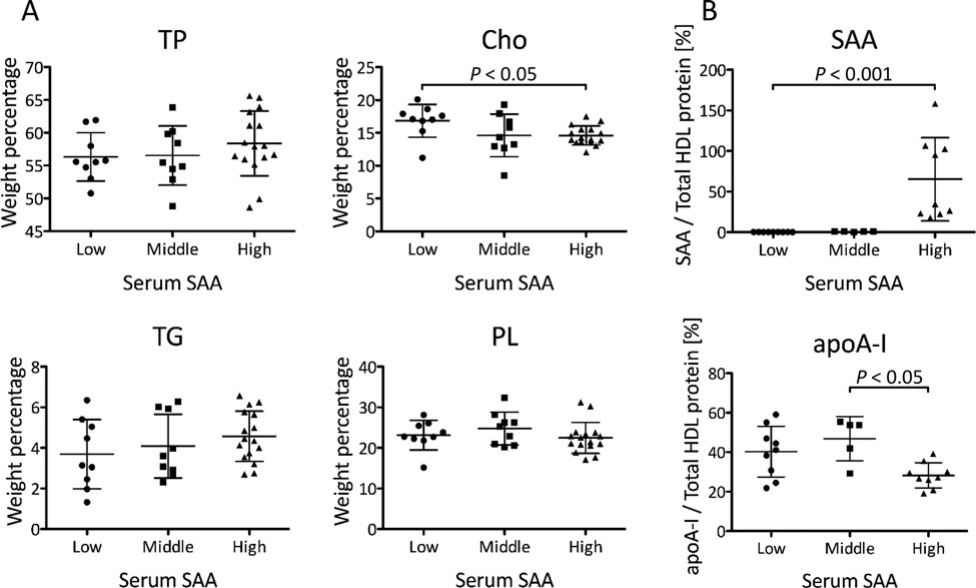
Composition of HDL obtained from the patients HDL samples were isolated from the patients with low SAA levels (Low, SAA ≤ 8 μg/ml), middle SAA levels (Middle, 8 < SAA ≤ 100 μg/ml) and high SAA levels (High, SAA > 100 μg/ml), and compared in terms of protein and lipid compositions. (**A**) Weight percentage of each component against total amount of protein, Cho, TG and PL in HDL was indicated (Low; *n*=9, Middle; *n*=9, High; *n*=16). (**B**) Weight percentage of SAA and apoA-I against total protein in HDL was indicated (Low; *n*=9, Middle; *n*=5, High; *n*=9). The samples with SAA or apoA-I levels lower than the minimum detectable were excluded. Data are shown as the mean ± S.D.

**Figure 4:**
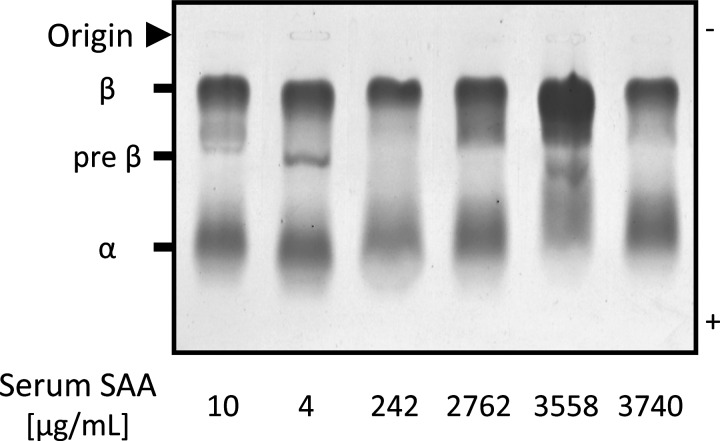
Surface charge of HDL obtained from the patients Sera from six patients were analysed by agarose gel electrophoresis followed by staining with Fat Red 7B.

**Figure 5:**
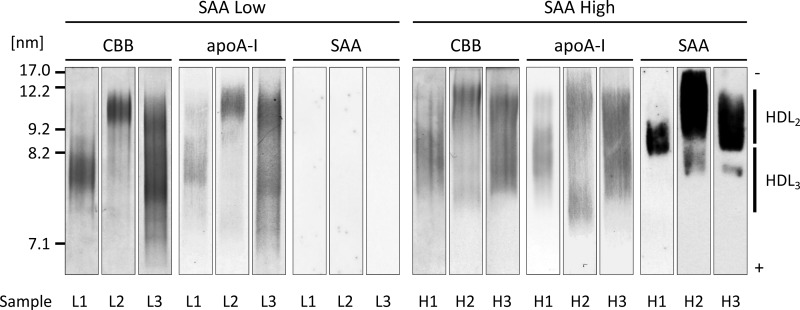
Distribution of SAA in HDL obtained from the patients HDL isolated from three patients from the Low SAA group (L1–3) and High SAA group (H1–3), respectively, were analysed by non-denaturing gel electrophoresis and visualized by CBB staining and by western blotting using anti-apoA-I antibody and anti-SAA antibody. These are representative patterns of HDL obtained from X and Y patients from Low SAA and High SAA groups, respectively. Serum SAA concentrations of the patients L1, L2, L3, H1, H2 and H3 were 6.0, 3.0, 3.0, 557, 3558 and 2762 μg/ml, respectively.

Firstly, we investigated the composition of HDL obtained from the patients by measuring the concentrations of TP and lipids. The ratio of cholesterol to total mass (protein, Cho, TG and PL) was significantly lower in the High SAA group than in the Low SAA group (*P*<0.05) (Figure 3A). However, there were no significant differences in other components (TP, TG and PL) among the three groups (Figure 3A). The ratio of SAA to total HDL protein mass was significantly higher (*P*<0.001) in the High SAA group than in the Low SAA group (Figure 3B). In contrast, the ratio of apoA-I to total HDL protein mass was lower (*P*<0.05) in the High SAA group than in the Middle SAA group. Secondly, to analyse the surface charge of HDL, serum samples of the patients from the three groups were subjected to the agarose gel electrophoresis. The relative electrophoretic mobilities of HDL were slightly reduced in the patients from the High SAA group compared with the patients from the Low SAA group (Figure 4). The degree of mobilities indicated an inversely proportional relationship to SAA levels. Finally, we estimated HDL particle size by non-denaturing gel electrophoresis. Various HDL particle sizes were observed in both Low and High SAA groups, regardless of serum SAA levels (Figure 5). The apparent amount ratios of HDL_2_ to HDL_3_ also varied among the patients. In contrast, SAA was identified in large HDL particles of patients from the High SAA group, but not in the small HDL particles, unlike for reconstituted SAA-HDL *in vitro*. HDL samples obtained from the Middle SAA group patients also showed a similar pattern to those collected from the High SAA patient group, even though extremely faint bands of SAA were obtained.

### Antioxidant ability of HDL

**Figure 6:**
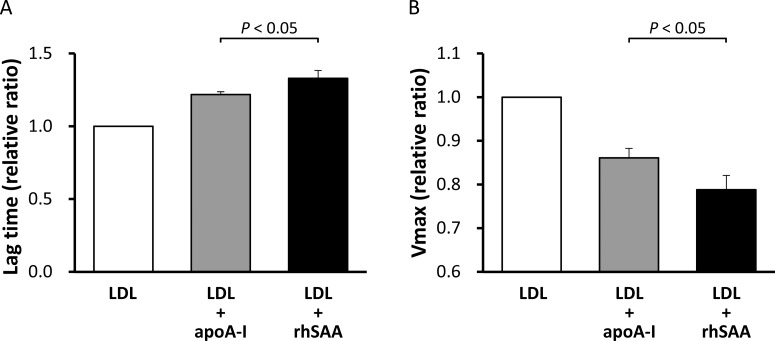
Antioxidant ability of rhSAA and apoA-I Antioxidant abilities of rhSAA and apoA-I were determined as described in the ‘Materials and Methods’ section. The values were expressed as the mean of the lag time (**A**) and *V*_max_ (**B**) relative to those of LDL alone, defined as 1. Bars represent S.D. from triplicate experiments.

**Figure 7:**
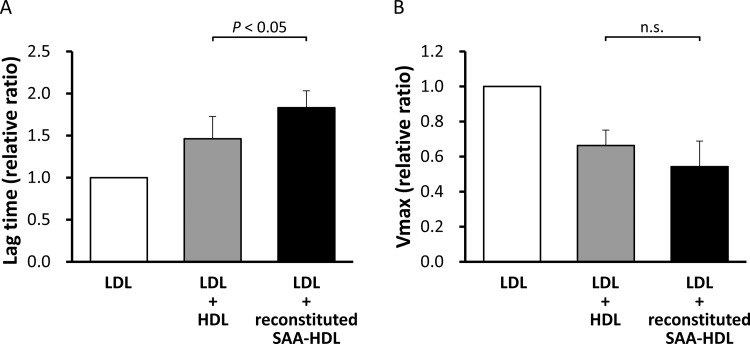
Antioxidant ability of reconstituted SAA-HDL and normal HDL HDLs obtained from healthy subjects (final HDL-Cho of 25 mg/dl) were incubated with or without rhSAA (final concentration of 1 mg/ml) for 60 min at room temperature. The mixtures were then ultracentrifuged at the density of 1.210 g/ml to remove unattached SAA. HDLs in top fractions were defined as reconstituted SAA-HDL (incubated with rhSAA) and normal HDL (incubated without rhSAA). Antioxidant abilities of both HDLs were determined as described in the ‘Materials and Methods’ section. The values were expressed as the mean of lag time (**A**) and *V*_max_ (**B**) relative to those of LDL alone, defined as 1. Bars represent S.D. from four separate experiments. n.s., not significant.

**Figure 8:**
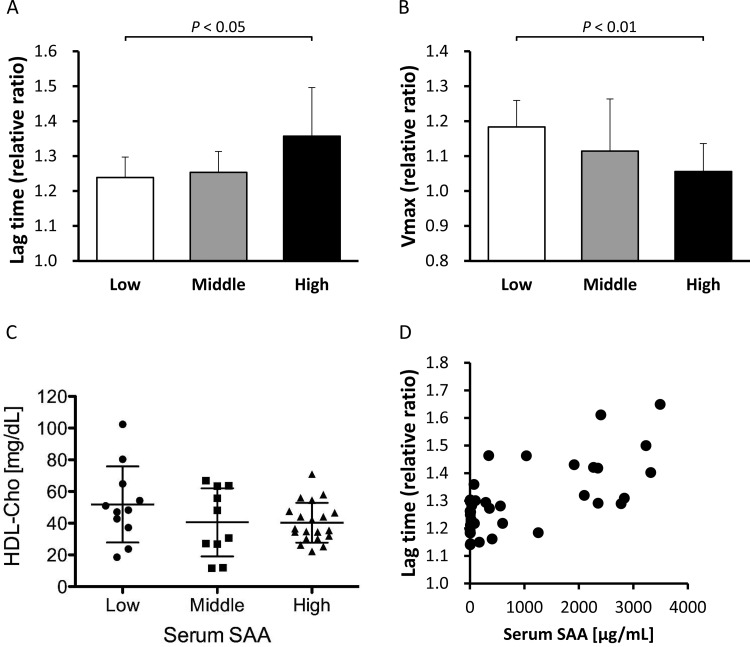
Antioxidant ability of HDL obtained from the patients Antioxidant abilities of HDLs obtained from the patients, stratified into three groups according to the concentration of serum SAA, Low (SAA ≤ 8 μg/ml, *n*=11), Middle (8 < SAA ≤ 100 μg/ml, *n*=10) and High (SAA > 100 μg/ml, *n*=20), were determined as described in the ‘Materials and Methods’ section. The values were expressed as the mean of lag time (**A**) and *V*_max_ (**B**) relative to those of LDL alone, defined as 1. Bars represent S.D. HDL-Cho levels in three groups (**C**) were also compared. Correlation between relative lag time and serum SAA levels (**D**) was analysed in all samples of three groups.

To investigate the effect of apoA-I displacement caused by SAA on the antioxidant ability of HDL, we compared the relative lag time and *V*_max_ between rhSAA and apoA-I. The mean ± S.D. for relative lag time of rhSAA was 1.33±0.05 whereas that for apoA-I was 1.22±0.02 (*P*<0.05) (Figure 6A). The relative *V*_max_ of rhSAA was 0.79±0.03 whereas that of apoA-I was 0.86±0.02 (*P*<0.05) (Figure 6B). These results indicate that rhSAA has a higher antioxidant ability than apoA-I against LDL oxidation. Subsequently, the antioxidant abilities of reconstituted SAA-HDL and normal HDL were determined. The relative lag time was significantly higher in reconstituted SAA-HDL (1.83±0.20) than in normal HDL (1.46±0.27) (*P*<0.05) (Figure 7A). Although there was no significant difference, the relative *V*_max_ tended to be lower in reconstituted SAA-HDL (0.54±0.15) than in normal HDL (0.66±0.09) (Figure 7B), consistent with the results obtained with the rhSAA protein. Moreover, we estimated the antioxidant ability of HDL from the patients of the three groups separately. The relative lag times for the Low, Middle and High SAA groups were 1.24±0.06, 1.25±0.06 and 1.36±0.14, respectively (*P*<0.05) (Figure 8A), and the relative *V*_max_ were 1.18±0.08, 1.11±0.15 and 1.06±0.08, respectively (*P*<0.01) (Figure 8B). HDL-Cho levels in the three groups were 51.8±24.0, 40.6±21.5 and 40.3±12.5 mg/dl, respectively (Figure 8C). Consequently, these results indicate that HDL from the High SAA group had the highest antioxidant ability despite displaying lower plasma HDL-Cho levels. Moreover, serum SAA levels were correlated with relative lag times in all samples (*r*=0.554, *n*=41, *P*<0.001) (Figure 8D).

## DISCUSSION

SAA concentrations increase in human subjects with various acute and chronic inflammatory conditions, and most of the SAA released from the liver is bound to HDL. Since HDL plays anti-atherosclerotic roles through apoA-I, investigating apoA-I displacement caused by SAA and its effects on HDL functions would provide important information regarding the relation between inflammation and atherosclerosis. Our study showed similar results to those obtained in previous reports, demonstrating that rhSAA was bound to HDL particles and released apoA-I from HDL *in vitro* (Figure 1); however, the HDL particle size was not significantly affected (Figure 2) possibly because only a small amount of SAA attached to HDL. Indeed, SAA contained approximately 17% apoA-I in reconstituted SAA-HDL, corresponding to the amount found in a chronic inflammation configuration, with remodelling HDL levels, instead of a severe inflammation situation. The features of reconstituted SAA-HDL prepared in the present study were similar to those used in a previous report [1], and SAA was bound selectively to HDL_3_, especially the smallest one. In the sample containing rhSAA alone, the band of SAA obtained from non-denaturing gel electrophoresis was observed at the position corresponding to a particle size of approximately 5 nm. This size is too large to correspond to the molecular mass of SAA, 11.7 kDa, suggesting that SAA aggregates in the buffer due to its hydrophobicity.

In contrast, various patterns of HDL remodelling were observed in the patients with severe inflammation. Serum SAA levels in the High SAA group were widely distributed, 170–3490 μg/ml, therefore, there was a large variability in the content ratio of SAA in HDL (Figure 3B). Consistent with our *in vitro* study, the ratio of apoA-I to total HDL protein mass in the High SAA group decreased, probably due to apoA-I displacement caused by SAA. Furthermore, HDL obtained from the patients showed a propensity to decrease relative cholesterol content proportional to SAA concentrations, whereas no significant differences in other components, such as proteins, TG and PL, were observed among the three groups (Figure 3A). Lecithin cholesterol acyltransferase (LCAT), which is activated by apoA-I, converts free cholesterol to cholesteryl esters (CE) in HDL. Lower cholesterol levels may reflect a reduction in CE levels in the lipid core because of decreased LCAT activity, resulting from apoA-I displacement caused by SAA [23].

On analysis of agarose gel electrophoresis patterns, the increased SAA levels induced a decrease in HDL mobility (Figure 4). This is probably because the isoelectric point of SAA attached to HDL is higher than that of apoA-I released from HDL. In fact, the isoelectric point of SAA1 is pI 6.1 whereas that of apoA-I is pI 5.75 [24,25], which means that the negative charge of SAA-HDL is lower than that of normal HDL. By non-denaturing gel electrophoresis, SAA was detected in both HDL_2_ and HDL_3_ obtained from the patients (Figure 5), whereas the result obtained *in vitro* showed that SAA existed in the extremely small reconstituted HDL particles. These results suggest that SAA is bound to small HDL_3_, which might then be converted to large particles with a size similar to that of HDL_2_, *in vivo*. In addition, HDL including SAA, especially small particles, would be quickly catabolized and removed from the circulation during inflammatory states [26–28]. Further experiments are required to elucidate these mechanisms. As indicated in the representative data (Figure 5), most of the SAA-HDL samples (*n*=13), analysed by western blotting, obtained from patients of the High SAA group were larger than 8.0 nm, implying that SAA might be easily bound to HDL_3_ and more rapidly catabolized than when bound to large HDL. SAA is known to generate HDL through ABCA1, similar to apoA-I [17]. This suggests that the various sizes of SAA-HDL observed in the patients are generated from nascent SAA-HDL, mimicking normal HDL metabolism. In other words, the displacement of apoA-I from HDL particles by SAA, could not be the main route leading to the construction of SAA-HDL in the circulation.

Antioxidant abilities were compared between rhSAA and apoA-I. Surprisingly, rhSAA extended relative lag time by 9% and reduced *V*_max_ by 8% compared with apoA-I (Figures 6A and 6B). Moreover, reconstituted SAA-HDL also prolonged relative lag time by 25% and decreased *V*_max_ by 18% compared with normal HDL (Figures 7A and 7B). Similarly, HDL obtained from High SAA group patients prolonged relative lag time in the oxidation profile (Figure 8A), indicating enhanced antioxidant ability. It was also confirmed by that serum SAA levels were correlated with relative lag times in all samples (Figure 8D). In general, HDL-Cho levels in patients with severe inflammation are lower than that in normal subjects, because of accelerated catabolism of SAA-HDL [1,29]. HDL-Cho concentrations within the High SAA group were 22% lower compared with those observed within Low SAA group (Figure 8C). Antioxidant abilities of HDL samples obtained from the High SAA group were 10% higher than that of those obtained from the Low SAA group (Figures 8A and 8B). This suggests a compensatory role of SAA for antioxidant ability of HDL, however, the correlation in percentages between a decrease in HDL-Cho and an increase in antioxidant ability, and prolonged relative lag time, is not obvious. Consequently, it is not clear whether absolute antioxidant ability in the patient is preserved or enhanced during inflammation. Furthermore, it is reported that SAA also binds to LDL [30]. To determine antioxidant ability, we used LDL obtained from healthy volunteers, and not from any of the patients; therefore, the effect of SAA-containing LDL on antioxidant ability was not established here. It would be necessary to use LDL and HDL samples from the same patient to establish a physiologically significant antioxidant ability comparison.

## CONCLUSION

Our data suggest that SAA displaces apoA-I from HDL, causing changes in its features and composition during inflammation. Notably, elevated SAA levels in serum leads to enhanced antioxidant ability of HDL. This is the first study to demonstrate that SAA promotes antioxidant ability. Although an analysis of a larger number of subjects with chronic and acute phase inflammation is needed, our results provide new information about the relation between inflammation and atherosclerosis. Further studies are required to understand the physiological and overall effects of SAA on the functions of the anti-atherogenic lipoprotein, HDL.

## Abbreviations

ABCA1: ATP-binding cassette transporter A1
apoA-I: apolipoprotein A-I
APR: acute phase response
CE: cholesteryl ester
CHD: coronary heart disease
HDL: high-density lipoprotein
HDL-Cho: HDL-cholesterol
LCAT: lecithin cholesterol acyltransferase
LDL: low-density lipoprotein
PL: phospholipid
RCT: reverse cholesterol transport
rhSAA: recombinant human SAA
SAA: serum amyloid A
SAA-HDL: SAA-containing HDL
TG: triacylglycerol
TP: total protein
